# Effect of Waste MDF-Derived Carbonisate Morphology on the Dielectric Stability of Epoxy-Based Composites

**DOI:** 10.3390/polym18162020

**Published:** 2026-08-20

**Authors:** Agata Wieczorska

**Affiliations:** Faculty of Marine Engineering, Gdynia Maritime University, Morska St. 81-87, 81-225 Gdynia, Poland; a.wieczorska@wm.umg.edu.pl

**Keywords:** epoxy composites, polymer composites, waste-derived carbon filler, MDF carbonisate, dielectric properties, electrical stability, microstructure, particle size effect, sustainable materials

## Abstract

This paper presents the next stage of research on epoxy-glass composites modified with carbonisate obtained from the pyrolysis of waste MDF boards. Previous studies focused on assessing the mechanical properties of the developed material, while the aim of this work was to expand its characterization to include assessment of electrical properties and to determine the potential use of carbonisate as a functional filler in electrically insulating composites. The effect of carbonisate particle size (<500, <1000, and <1500 µm), carbonisate content (5% and 7.5% by weight), and resin-to-reinforcement ratio on the surface and volume resistivity of epoxy-glass composites was analysed. The microstructure of the materials was assessed using optical microscopy and scanning electron microscopy (SEM). To quantitatively assess changes in electrical insulating properties, we used logarithmic resistivity analysis and our own dielectric destabilization index (D), which serves as a comparative engineering index. The obtained results showed that increasing the carbonisate particle size leads to a gradual decrease in the electrical insulating properties of the composites and an increase in their structural heterogeneity. The highest dielectric destabilization index value was obtained for the composite containing 7.5% carbonisate with a fraction <1500 µm (D = 0.403). Microstructural observations confirmed the presence of agglomerates, micropores, and local structural discontinuities, which can reduce the electrical resistivity of the tested materials. The conducted studies confirmed the possibility of using carbonisate obtained from waste MDF (Medium-Density Fibreboard) boards as a functional filler in epoxy-glass composites. The obtained results constitute the next stage of comprehensive characterization of the developed material and indicate that the appropriate selection of the carbonisate morphology and the composition of the composite may enable shaping its electrical insulating properties depending on the planned application.

## 1. Introduction

The dielectric properties of polymer composites have become an important area of materials research due to the possibility of controlling electrical conductivity, insulation stability, and the functional performance of materials [1,2,3]. Particular attention has been focused on epoxy composites modified with carbon-based fillers, as the presence of conductive phases may significantly affect the electrical, thermal, and mechanical properties of the material [4,5,6]. Previous studies have demonstrated that both the type and content of the filler can influence the improvement of selected functional properties as well as contribute to the destabilization of the dielectric behaviour of epoxy composites [7,8].

According to the literature, the dielectric properties of composites depend not only on the type of filler used, but also on particle size, the degree of filler dispersion, and the interactions between the conductive phase and the polymer matrix [9,10,11]. The presence of carbon-based additives may lead to the formation of local conductive pathways, microstructural changes, and a reduction in the electrical resistance of the material [10,11,12]. At the same time, increasing structural heterogeneity of the composite may promote the formation of pores, microcracks, and local regions of stress concentration and electrical conductivity [11,12,13].

Another important research issue is the possibility of tailoring the electrical properties of composites through the control of conductive network architecture and the appropriate selection of hybrid filler systems [13,14]. As reported in studies on dielectric materials, changes in the geometry and distribution of conductive particles may lead to the destabilization of insulation parameters, particularly when the filler is non-uniformly dispersed within the polymer matrix [15].

In recent years, growing attention has been devoted to the use of waste-derived materials as functional modifiers of polymer composites. Of particular interest are carbonisates obtained from lignocellulosic waste, which may serve as an alternative to conventional carbon fillers while simultaneously reducing the production costs of composite materials. In study [16], it was demonstrated that carbon-based additives may affect the wettability of reinforcing fibres and the quality of the fibre–matrix interface. In turn, the results presented in [17] indicated that carbon particles incorporated into thermosetting composites may alter the microstructure of the material and influence its mechanical and functional properties. Furthermore, study [18] emphasized that fillers may significantly affect the mechanical, thermal, and electrical properties of polymer materials, especially when the distribution of phases within the composite structure is non-uniform.

Common inorganic and mineral powder materials used as fillers in polyolefin composites include calcium carbonate, glass powders, carbon-based fillers, metal powders (e.g., copper and aluminium), talc, mica, wollastonite, various forms of silica, and clays. The diverse morphology and structure of these additives may lead to the formation of agglomerates within the polymer matrix, thereby affecting the microstructural homogeneity and mechanical properties of the resulting composite materials [19,20,21,22].

The modification of epoxy resins with various fillers is one of the most widely applied approaches for tailoring the functional properties of polymer composites. In addition to improving mechanical and thermal performance, appropriately selected fillers may also influence the electrical properties of materials, including conductivity, electrical resistance, and dielectric stability [23,24,25]. Particular attention has been devoted to materials characterized by a highly developed specific surface area and diverse particle morphology, as their presence may induce microstructural changes and promote the formation of local conductive pathways within the composite. These effects depend not only on the type of filler employed but also on its particle size, shape, degree of dispersion, and interactions with the polymer matrix [26,27,28,29].

In previous studies conducted by the authors, the influence of carbonisate obtained through the pyrolysis of waste MDF boards on selected functional properties of epoxy–glass composites was investigated. The results demonstrated that this material can effectively modify both the structure and the mechanical properties of the composites. In a study focused on the analysis of mechanical performance using Kolmogorov–Sinai metric entropy, carbonisate was shown to affect the strength parameters of the composites as well as the deformation dynamics of the material under static loading conditions. Furthermore, the presence of pores, microcracks, and local structural discontinuities associated with the incorporation of the filler was identified [30].

Subsequent studies demonstrated that MDF-derived carbonisate may influence both the hardness and impact strength of epoxy–glass composites, with the observed effects depending on filler content, particle size, and the proportion of the polymer matrix. It was also shown that this material can serve as a functional waste-derived filler capable of modifying the mechanical properties of composites [31]. Moreover, the analysis of the functional performance of composites using Kolmogorov–Sinai metric entropy confirmed the suitability of MDF carbonisate for tailoring the operational properties of composite materials [32]. The obtained results showed that the carbonisate content, its morphology, and the proportion of matrix and reinforcement significantly influenced the mechanical properties of the developed composites. These results formed the basis for expanding the research program to include the assessment of the electrical properties of the materials.

Since composites intended for structural applications often have to simultaneously meet requirements for mechanical strength and electrical insulating properties, it has become necessary to determine the effect of the same technological parameters on the electrical properties of the material.

The practical significance of these findings is further supported by the development of a patented solution concerning the manufacture of carbonisate-containing composite materials and their potential applications.

Despite the growing interest in biochar and carbon-based materials obtained through the thermochemical conversion of biomass, the use of carbonisate derived from waste MDF boards as a filler for polymer composites remains largely unexplored. In particular, the influence of carbonisate particle size on the dielectric properties of epoxy composites and the mechanisms responsible for their electrical stability have not yet been thoroughly investigated.

This issue is of considerable practical importance because waste MDF boards represent a difficult-to-manage waste stream due to the presence of synthetic resins and technological additives that complicate conventional recycling processes. One of the most promising approaches for their utilization is pyrolysis, which enables the conversion of waste into a carbon-rich carbonisate that may serve as a valuable secondary raw material in accordance with circular economy principles.

Previous studies have demonstrated that carbonisate obtained from waste MDF boards can effectively modify the structure and mechanical properties of epoxy–glass composites. These findings indicate the potential of this material as a functional waste-derived filler. Consequently, it became necessary to determine how the presence of carbonisate affects the electrical properties of composites, which are equally important as mechanical properties in many engineering applications.

The aim of this study was to evaluate the effect of carbonisate particle size, obtained from waste MDF boards, on the dielectric stability and microstructure of epoxy–glass composites. Particular attention was paid to the relationship between filler particle size, its distribution within the polymer matrix, the formation of local structural heterogeneities, and changes in the insulating properties of the material.

The novelty of this work lies in determining the influence of MDF-derived carbonisate, obtained through the pyrolysis of waste MDF boards, on the dielectric properties of epoxy–glass composites and in correlating changes in electrical stability with the observed microstructure of the material. An additional innovative aspect of the study is the application of an original dielectric destabilization index (D), proposed as a comparative engineering index, enabling the quantitative assessment of changes in the electrical insulating properties of the tested composites and facilitating the interpretation of the obtained results.

## 2. Materials and Methods

Epidian 6 (CIECH Sarzyna S.A., Nowa Sarzyna, Poland) epoxy resin was used as the epoxy matrix and glass mat as the composite reinforcement. Epidian 6 is a clear, viscous epoxy resin characterized by an epoxy equivalent of 185–196 g/eq, an epoxy value of 0.510–0.540 mol/100 g, and a viscosity of 10,000–15,000 mPa·s at 25 °C. The resin contains a minimum of 99.0% non-volatile matter. Z-1, an amine-based hardener recommended for Epidian 6, was used as the curing agent. The hardener is a clear, light-yellow liquid with a density of 0.978–0.983 g/cm^3^ at 20 °C and a minimum amine value of 1100 mg KOH/g. The resin and Z-1 hardener were mixed at a weight ratio of 100:13, in accordance with the manufacturer’s recommendations. A carbonisate material derived from waste MDF boards was used as a modifier. The carbonisate material underwent a grinding process and particle size segregation.

Prior to composite manufacturing, the MDF-derived carbonisate was characterized in terms of its elemental composition and selected physicochemical properties. The material contained 79.17 wt.% carbon, 4.43 wt.% nitrogen, and 2.99 wt.% hydrogen. The ash content was 6.95 wt.%, while the moisture and volatile matter contents were 1.6 wt.% and 14.6 wt.%, respectively. These results confirmed the carbon-rich character of the material obtained from the pyrolysis of waste MDF boards. Subsequently, the carbonisate was mechanically ground and sieved into fractions below <500 μm, <1000 μm and <1500 μm, which were used for composite preparation. The carbonisate content in the composites was 5% and 7.5%. In addition, reference samples containing no carbonisate material were prepared. The composites were prepared for three matrix-to-reinforcement ratios: 65/35, 60/40 and 70/30.

The appropriate amount of carbonisate was added to the liquid epoxy resin and mechanically mixed until a dispersion of particles as homogeneous as possible was obtained.

The Z-1 hardener was then added to the resin–carbonisate mixture at the specified weight ratio (100:13) and thoroughly mixed.

The mixture prepared in this way was applied to a glass mat, forming composites with the specified resin-to-fibre ratio. The curing process was carried out under laboratory conditions in accordance with the technical specifications for Epidian 6 resin. After curing, the samples were conditioned under laboratory conditions prior to the dielectric tests.

The composites were manufactured using the hand lamination method. The carbonisate preparation process, composite manufacturing technology, curing parameters, and detailed characteristics of the materials used were described in the authors’ previous publications [30,31,32]. An identical technological procedure was used in this study, which ensured the comparability of the obtained results and enabled the material characterization to be extended to include an assessment of its electrical properties.

Table 1 summarises the composition of the composite materials prepared using the hand-lay-up method, including the proportions of the individual components and the designations of the samples used.

The scope of the study included measurements of the surface and volume resistance of epoxy composites modified with MDF carbonisate.

The dielectric property tests were carried out in accordance with standard [33] in an accredited laboratory. The measurements were taken at a temperature of 22.7 °C and a relative humidity of 48.6%. The samples were conditioned in a climatic chamber for 24 h at a temperature of 23 °C and a relative humidity of 50%. Surface and volume resistivity measurements were carried out using a METRISO meter (Gossen Metrawatt GmbH, Nürnberg, Germany), at a fixed test voltage of 500 V, at three points on the sample surface.

The uncertainty of resistance measurements was determined in accordance with the guidelines of document EA-4/02 “Evaluation of the Uncertainty of Measurement in Calibration” in an accredited laboratory. The values given are expanded uncertainties at a confidence level of 95% and a coverage factor (k = 2).

The measuring instrument used in this study had an upper measurement limit of 10^12^ Ω. Samples exhibiting resistance values above this limit were recorded as >10^12^ Ω, per the manufacturer’s specifications. For the purposes of logarithmic comparison and calculation of the dielectric destabilization index D, values exceeding the measurement range were conservatively assigned to the upper measurement limit (10^12^ Ω). Therefore, the calculated D values for these materials should be interpreted as minimum estimates of dielectric destabilization and not as absolute dielectric parameters.

**Figure 1:**
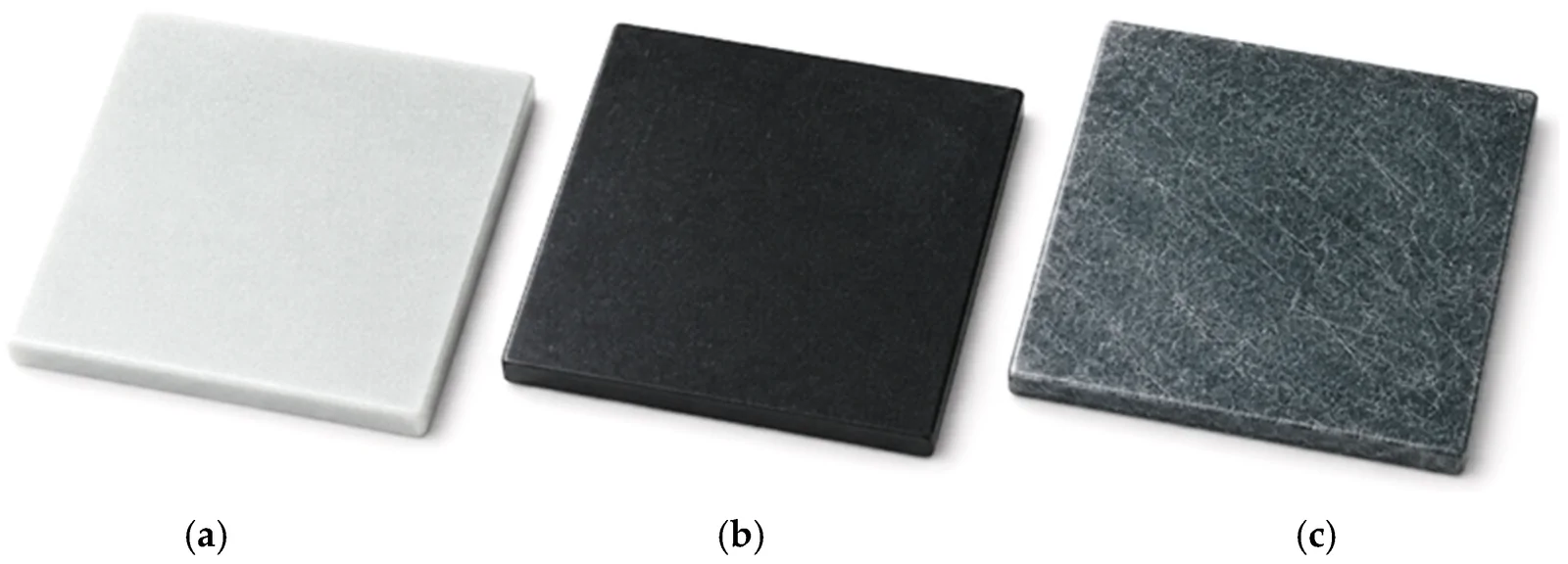
Composite samples with dimensions of 200 × 200 mm from the 60/40 group: (**a**) reference sample Y, (**b**) sample G containing carbonisate with a particle size fraction of <500 µm, and (**c**) sample I containing carbonisate with a particle size fraction of <1000 µm.

Twenty-one composite specimens measuring 200 × 200 mm were prepared for the tests. The selected specimens are shown in Figure 1.

Three measurements were taken for each sample; Table 2 summarises the average values of the three measurements of surface and volume resistivity.

### Data Processing and Logarithmic Analysis

Three independent measurements of surface and volume resistivity were carried out for each sample. The arithmetic mean and standard deviation were calculated on the basis of the results obtained.

The average resistance value was determined according to the relationship:

(1) $$\bar{x}=\frac{1}{n}\sum_{i=1}^{n}x_{i}$$

where: $\bar{x}$—arithmetic mean, $x_{i}$—single measurement result, n—number of measurements.

The standard deviation is calculated according to the formula:

(2) $$SD=\sqrt{\frac{\sum (x_{i\;}-\bar{x}{)}^{2}}{n-1}}$$

where: SD—standard deviation, $\bar{x}$—arithmetic mean, $x_{i}$—single measurement result, n—number of measurements.

Due to the very wide range of resistance values obtained, spanning several orders of magnitude, the results were additionally analyzed on a logarithmic scale. Using logarithms enabled a quantitative comparison of changes in dielectric properties between the tested samples and an assessment of the degree of destabilization of the composites’ insulating properties.

The decimal logarithm of resistance was used for the analysis:

(3) $$\log_{10}\left(R\right)$$

where: R—resistance value expressed in ohms.

Logarithmic analysis is widely used in the study of the electrical properties of polymeric materials, as it allows for the comparison of changes in resistance occurring across different orders of magnitude.

Additionally, it should be emphasized that the proposed approach was based on three independent measurements for each sample and focused on evaluating the effects of carbonisate particle size and filler content relative to the corresponding reference composites. Owing to the manufacturing variability of composite materials and the inherent limitations of resistance measurements, the proposed methodology should be regarded primarily as a comparative tool for assessing trends in dielectric behaviour. Although it enables a quantitative comparison of the investigated systems, it does not constitute a comprehensive dielectric characterization.

In order to quantitatively compare changes in dielectric properties relative to the reference samples, the resistance decay parameter was calculated:

(4) $$∆\log R=\log_{10}(R_{ref})-\log_{10}(R_{i})$$

where: $R_{ref}$—resistance of the reference sample, $R_{i}$—resistance of the sample under analysis.

The ΔlogR parameter quantifies the change in resistance in terms of orders of magnitude.

To compare the degree of dielectric property destabilization, the dielectric stability index was introduced:

(5) $$SI=\frac{R_{ref}}{R_{i}}$$

where: SI—dielectric stability index, $R_{ref}$—resistance of the sample under test, $R_{i}$—resistance of the reference sample.

Additionally, the dielectric destabilization index D was defined as the maximum logarithmic decrease in surface or volume resistance relative to the reference sample:

(6) $$D=max(∆\log R_{s},∆\log R_{v})$$

where: $R_{s}$—surface resistance, $R_{v}$—volume resistance.

The proposed dielectric destabilization index D is a comparative engineering index developed to quantitatively assess the impact of technological parameters on changes in the electrical insulating properties of the tested composites. It does not replace classical dielectric parameters such as permittivity, dielectric loss factor, or impedance spectroscopy results, but rather provides a tool supporting the comparative analysis of materials tested under identical experimental conditions.

## 3. Results

This chapter presents the results of investigations into the dielectric properties of epoxy-glass composites modified with MDF carbonisate material of varying particle sizes and content. The analysis included an assessment of surface and volume resistivity, a logarithmic analysis of electrical parameters, and a quantitative assessment of the degree of dielectric destabilisation in the tested materials. In addition, a comparative analysis was carried out of the influence of carbonisate particle size, filler content and composite composition on the stability of the insulating properties.

### 3.1. Surface and Volume Resistance of the Composites

**Figure 2:**
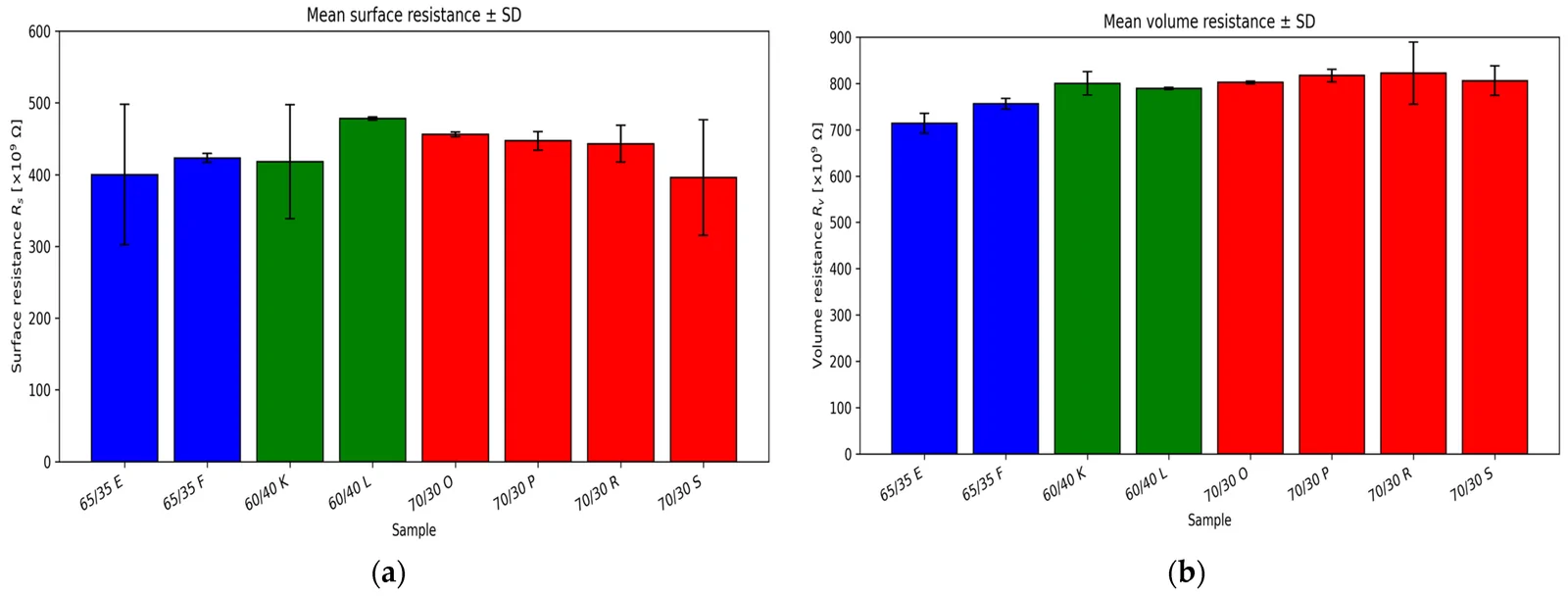
Average resistance value: (**a**) surface resistance $R_{s}$ ± SD, (**b**) volume resistance $R_{v}$ ± SD.

Table 3 presents the average values of the surface and volume resistivity of the tested composites, together with their standard deviations. The analysis of the effect of carbonisate particle size and composite composition on the dielectric properties is supplemented by graphs showing the variation of the analysed parameters, as presented in Figure 2.

In the case of surface resistance, the lowest value was recorded for sample 70/30 S (396 × 10^9^ Ω), whereas the highest value was observed for sample 60/40 L (478 × 10^9^ Ω). Sample 70/30 S also exhibited the highest standard deviation (80.6 × 10^9^ Ω), indicating greater heterogeneity in the material structure and a less uniform distribution of carbonisate particles. In contrast, samples 60/40 L and 70/30 O showed the lowest standard deviations, suggesting a more homogeneous microstructure.

Smaller differences were observed for volume resistance than for surface resistance. The measured values ranged from 714 × 10^9^ Ω (sample 65/35 E) to 822 × 10^9^ Ω (sample 70/30 R), indicating that the addition of carbonisate had a more pronounced effect on the electrical properties of the composite surface than on the bulk material.

Within individual composite groups, increasing the particle size from <1000 µm to <1500 µm generally resulted in a reduction in surface resistance, particularly for the 70/30 composites. In contrast, volume resistance remained relatively stable, suggesting that particle size affected mainly the surface electrical behaviour of the composites, while the bulk insulating properties changed only slightly.

### 3.2. Logarithmic Analysis and Dielectric Destabilization

The changes in dielectric properties were evaluated using logarithmic analysis of surface and volume resistivity together with the dielectric destabilization index (D).

The changes in the dielectric properties of the composites under investigation were analysed using logarithmic values of surface and volume resistivity. Based on the data obtained, the parameters ΔlogR, the dielectric stability index (SI) and the dielectric destabilisation index D were determined. To facilitate the interpretation of the results, a proposed engineering classification of the dielectric destabilization index (D) was developed and is presented in Table 4. This classification is intended solely as a comparative tool for the investigated material system.

Based on the classification presented, an assessment was carried out of the degree of dielectric destabilisation in the composites under investigation. The results for the logarithmic parameters and the assigned destabilisation classes are summarised in Table 5.

Analysis of the results presented showed that samples containing carbonisate with a particle size of <500 µm did not exhibit any significant destabilisation of their dielectric properties, and the D-index values remained at 0. As the particle size of the carbonisate increased, a gradual rise in the ΔlogR values and the dielectric destabilisation index was observed. The highest D = 0.403 was obtained for the 70/30 S sample, indicating the greatest reduction in insulating properties among all the materials analysed.

Greater changes were observed in surface resistance than in volume resistance, which may suggest that dielectric destabilisation occurred mainly in the surface layer of the composites. The results suggest that the particle size of the carbonisate material had a greater influence on the destabilisation of the dielectric properties than the filler content itself. However, it should be noted that the effects of particle size and filler content cannot be completely separated in the present experimental design. Therefore, the results suggest that particle size had a stronger influence than filler content within the investigated experimental range.

**Figure 3:**
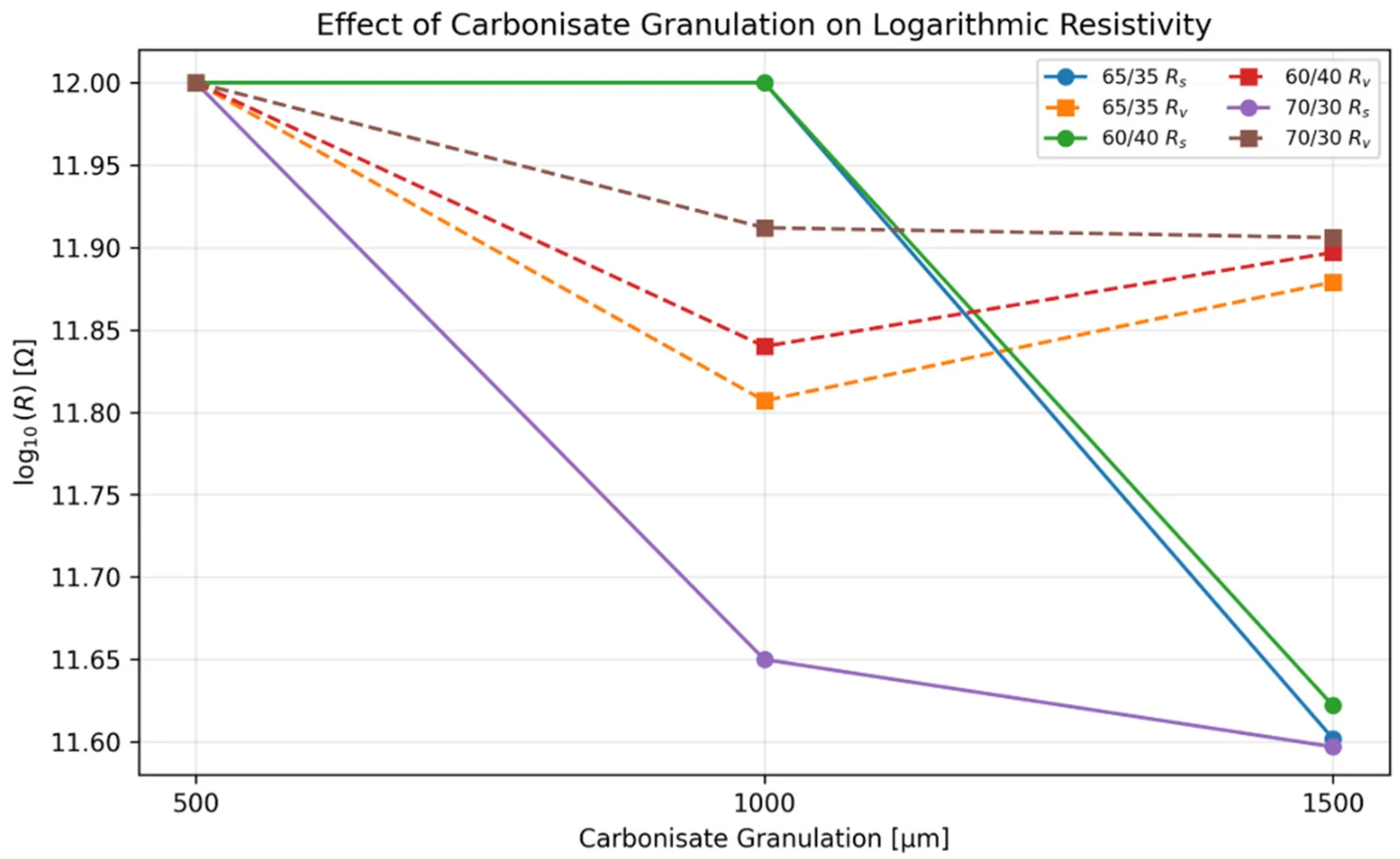
Effect of carbonisate particle size on the logarithmic values of surface and volume resistance of epoxy composites.

To better illustrate the effect of carbonisate particle size on changes in the dielectric properties of composites, the relationship between the logarithmic values of surface and volume resistivity and the particle size of the carbonisate is presented in Figure 3. Analysis of the graph allows for an assessment of the degree to which the insulating properties of the tested materials are destabilised as a function of the particle size of the filler used.

Logarithmic analysis showed that an increase in the particle size of the carbonisate resulted in a gradual decrease in the resistance of the composites under investigation. For all systems containing carbonisate with a particle size of <500 µm, no significant changes in dielectric properties were observed, and the log10 (R) values remained at around 12, corresponding to a resistance of the order of 10^12^ Ω. As the particle size increased to <1000 µm and <1500 µm, a progressive decrease in both surface and volume resistance was observed. The lowest logarithmic surface resistance was recorded for sample 70/30 S, indicating the strongest reduction in insulating performance.

In the case of volume resistance, the changes were less pronounced than for surface resistance, suggesting that dielectric destabilisation occurred mainly in the surface layer of the composites. The results obtained confirm that the granulation of the carbonisate material is a significant parameter influencing the insulating properties of the materials under investigation.

**Figure 4:**
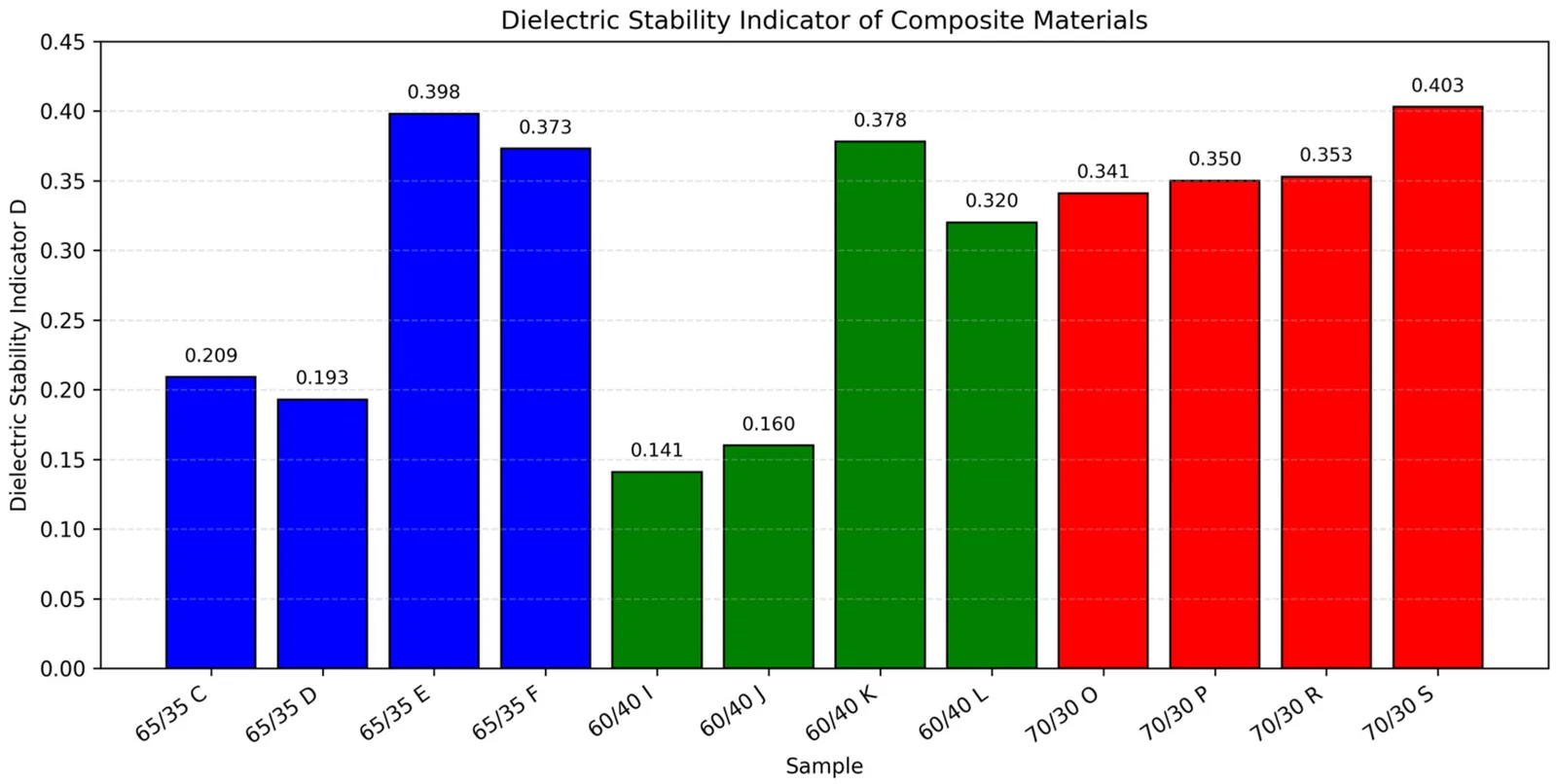
Values of the dielectric destabilization index D for the investigated epoxy composites.

The degree of destabilisation of the dielectric properties of the composites under investigation was assessed on the basis of the dielectric destabilisation index D, defined as the maximum decrease in the logarithmic value of the surface or volume resistivity relative to the reference sample. The values of the D index are shown in Figure 4.

The value of the dielectric destabilization index D increased with increasing carbonisate particle size. The highest level of destabilization was observed for sample 70/30 S (D = 0.403), indicating the greatest reduction in insulating properties among all analysed materials.

No significant destabilisation of the dielectric properties was observed in samples containing carbonisate with a particle size of <500 µm. The results obtained indicate a clear influence of carbonisate particle size on changes in the dielectric parameters of the composites under investigation.

### 3.3. Effect of Carbonisate Granulation on Dielectric Properties

To determine the effect of carbonisate particle size on the dielectric properties of the composites, the mean values of logarithmic parameters and the dielectric destabilization index were calculated for individual granulometric fractions of the carbonisate (Table 6).

An analysis of the mean values showed that an increase in the particle size of the carbonisate led to a gradual decrease in the resistivity of the composites and an increase in the mean value of the dielectric destabilisation index D. The greatest destabilisation was observed for a particle size of <1500 µm.

Mean values of logarithmic parameters and the dielectric destabilization index D for the investigated carbonisate contents are presented in Table 7. The analysis of the results enables evaluation of the effect of filler content on the stability of the insulating properties of the composites.

The results indicate that increasing the carbonisate content from 5% to 7.5% had a negligible effect on the dielectric properties of the composites. The mean dielectric destabilization index (D) remained almost unchanged (0.202 and 0.200, respectively), indicating that filler content had only a minor influence on dielectric stability. In contrast, particle size was found to be the dominant factor affecting the dielectric properties of the investigated composites.

Additionally, the influence of composite composition on the dielectric properties of the investigated materials was analysed by determining the mean parameter values for individual matrix–reinforcement systems (Table 8).

Among the investigated composite systems, the 70/30 composition exhibited the highest mean dielectric destabilization index (D = 0.241) and the lowest mean logarithmic surface resistance (log10(Rs) = 11.759), indicating the greatest susceptibility to dielectric destabilization and the formation of local conductive pathways. In contrast, the 60/40 composites showed the lowest mean D value (0.166), demonstrating the highest dielectric stability. Although the 70/30 system exhibited the highest mean logarithmic volume resistance (log10(Rv) = 11.940), the overall results suggest that the dielectric destabilization was more pronounced at the composite surface than in the bulk material.

**Figure 5:**
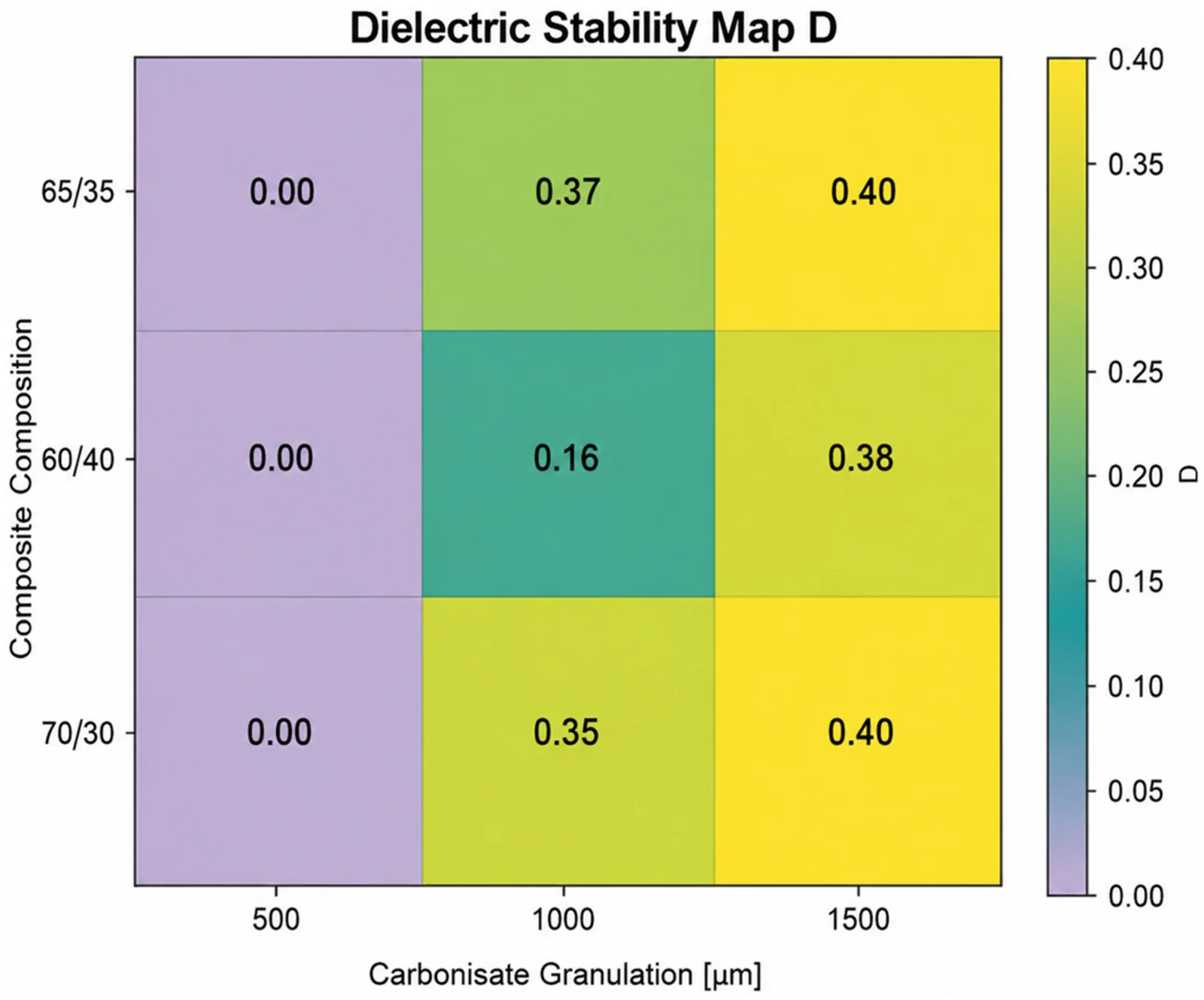
Map of the dielectric destabilization index D values for the investigated composites.

A map of the dielectric destabilization index D values is presented in Figure 5 to illustrate the influence of carbonisate particle size and composite composition on the stability of insulating properties. The obtained distributions enabled identification of the systems exhibiting the highest degree of dielectric destabilization.

Analysis of the dielectric destabilisation map revealed a progressive increase in the dielectric destabilisation index (D) with increasing carbonisate particle size. No dielectric destabilisation was observed for composites containing carbonisate with a particle size below 500 µm (D = 0), whereas the highest D values were obtained for materials containing carbonisate with a particle size below 1500 µm.

Among the investigated composite systems, the 70/30 composition exhibited the highest degree of dielectric destabilisation, reaching the maximum D value of 0.403 for the <1500 µm fraction. In contrast, the 60/40 composites showed the lowest destabilisation levels at both <1000 µm and <1500 µm particle sizes. These results confirm that carbonisate particle size is the dominant factor controlling dielectric stability, whereas the effect of composite composition is of secondary importance.

### 3.4. Optical Microscopy Analysis

**Figure 6:**
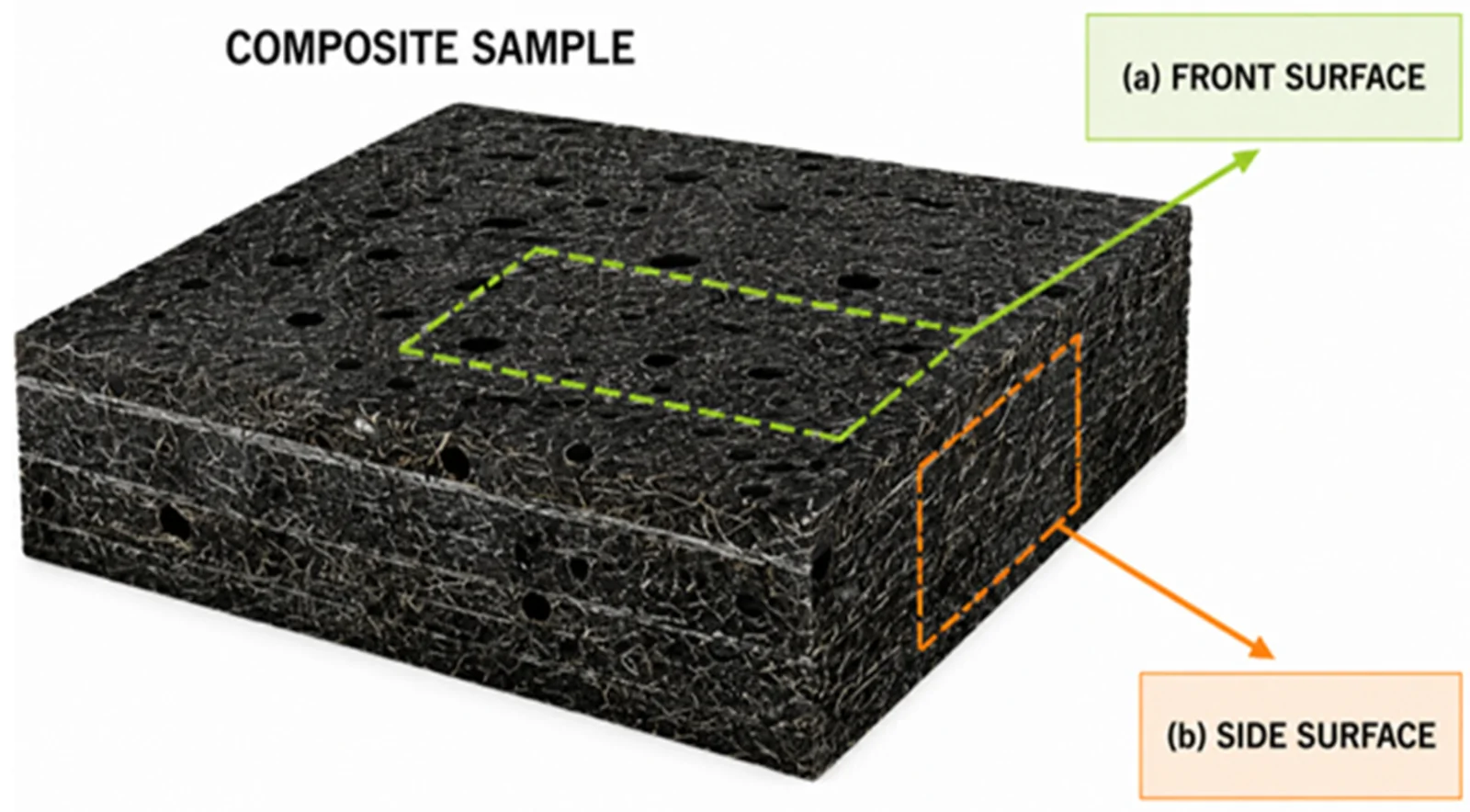
Scheme of the microscopic observation areas of epoxy–glass composites modified with MDF carbonisate: (**a**) front surface of the sample, (**b**) side surface of the sample.

In order to assess the microstructure and distribution of carbonisate in the composites, microscopic observations were carried out on the front and side surfaces of the samples. A diagram of the analysed areas is shown in Figure 6. The investigations were carried out using a Zeiss SmartZoom 5 digital industrial microscope (Carl Zeiss Microscopy GmbH, Jena, Germany) with a measurement accuracy of 1 × 10^−3^ mm. Observations of the front surface were carried out at a magnification of 300×, whilst a magnification of 100× was used for the side surface.

**Figure 7:**
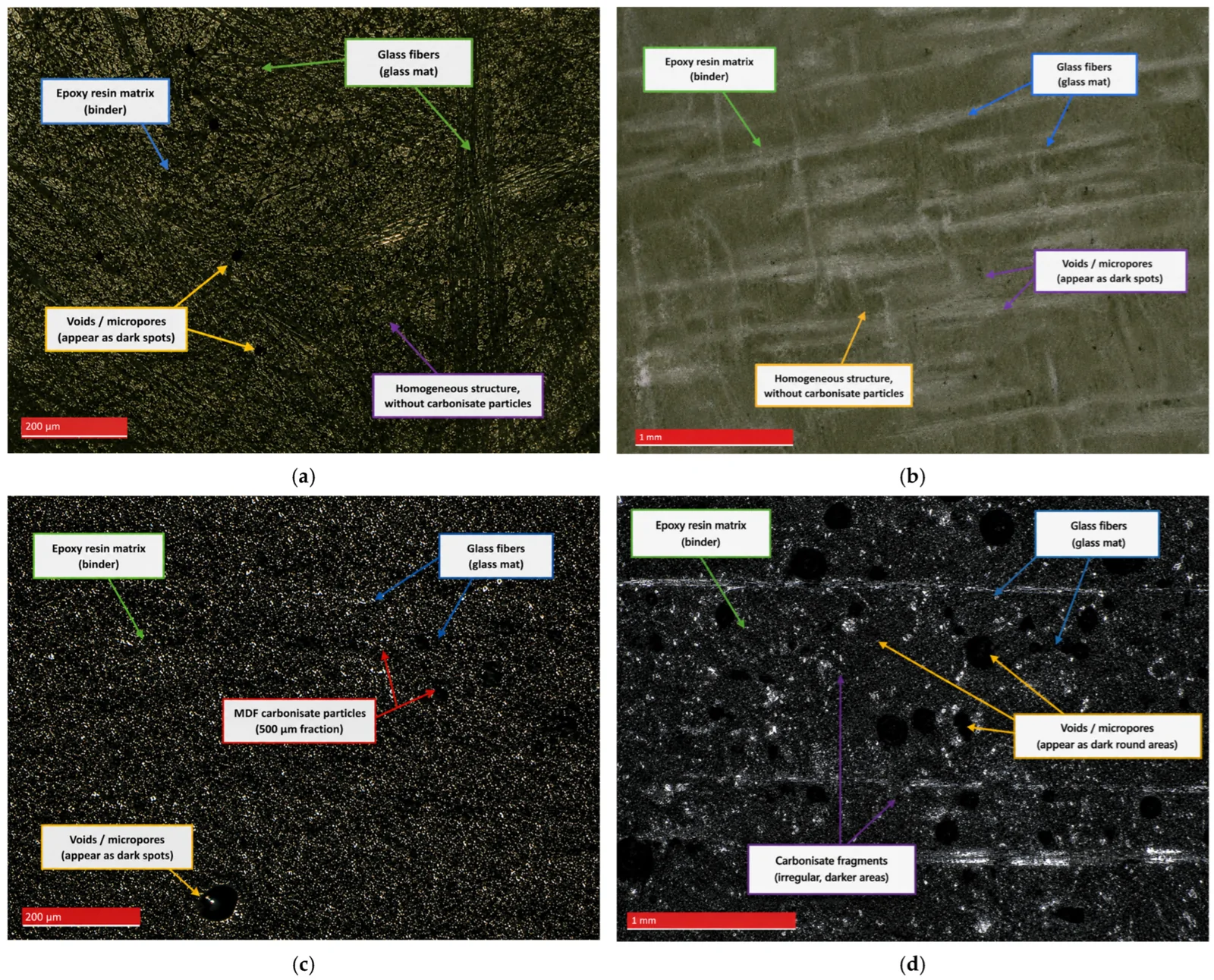

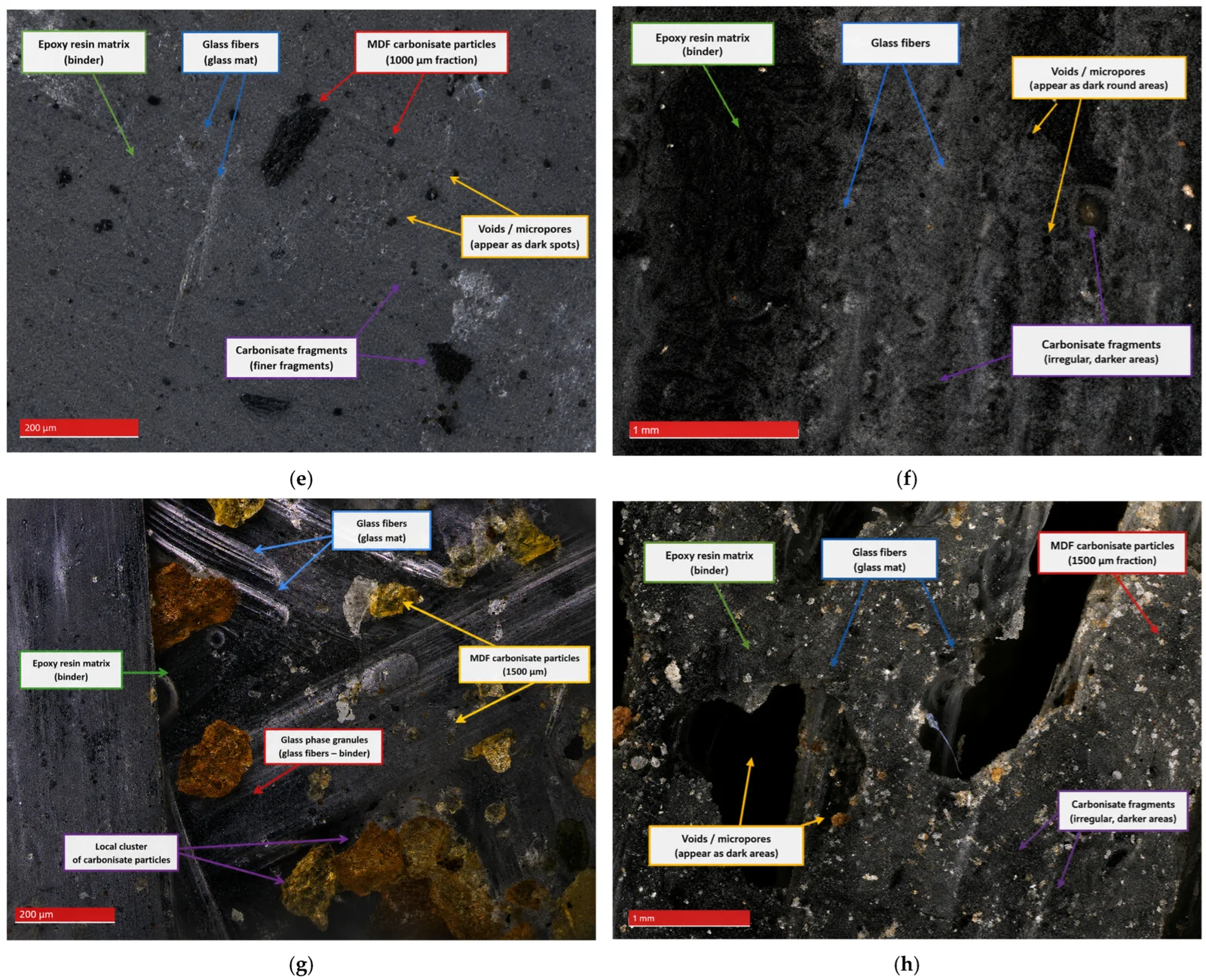
Microscopic images of the composites under investigation: (**a**,**b**) reference sample without carbonisate MDF, (**c**,**d**) composite containing carbonisate MDF with a particle size of <500 µm, (**e**,**f**) composite containing carbonisate MDF with a particle size of <1000 µm, (**g**,**h**) composite containing MDF carbonisate with a particle size of <1500 µm; (**a**,**c**,**e**,**g**) front surface, (**b**,**d**,**f**,**h**) side surface.

Examination of the front surface made it possible to assess the distribution of carbonisate material and local variations in the material’s structure. Analysis of the side surface, meanwhile, allowed for an assessment of the arrangement of the glass mat layers, the resin impregnation, and the presence of pores and microvoids in the cross-section of the composite, as presented in Figure 7.

In the reference sample without carbonisate, a relatively homogeneous epoxy matrix structure was observed, with a visible arrangement of glass fibres and isolated microvoids.

As the carbonisate particle size increased, the composites exhibited increasingly greater structural heterogeneity and the presence of larger filler agglomerates. In the case of the <500 µm fraction, the carbonisate was dispersed relatively evenly, whereas for particle sizes of <1000 µm and <1500 µm, larger irregular clusters of MDF carbonisate particles were visible.

For the sample containing carbonisate MDF with a <1500 µm particle size, the largest local clusters of filler and more distinct phase boundaries between the epoxy matrix, glass fibres and carbonisate MDF particles were observed. The presence of microvoids and local structural inhomogeneities may have contributed to the destabilisation of the dielectric properties of the composites under investigation.

The optical microscopy observations revealed differences in the microstructural homogeneity depending on the carbonisate particle size. The composites containing the smallest particles exhibited a more uniform filler distribution within the epoxy matrix. As the particle size increased, the occurrence of filler-rich regions and local agglomerates became more pronounced, indicating a gradual increase in structural heterogeneity. The largest particle fraction showed the highest tendency toward agglomeration and the formation of localized defects, which may affect the dielectric response of the composites.

The presented analysis of microscopic images is primarily qualitative in nature. The observed agglomerates, pores, and local structural discontinuities should be interpreted as features accompanying changes in dielectric properties rather than as a quantitatively verified causal mechanism. Nevertheless, the microscopic observations remain consistent with the trends identified in the resistance analysis and the dielectric destabilization index (D).

### 3.5. SEM Analysis of Carbonisate Morphology

The MDF-derived carbonisate used in this study contained 79.17 wt.% carbon and 6.95 wt.% ash, confirming its carbon-rich character. These physicochemical characteristics provide an important background for the interpretation of the observed morphology and structural heterogeneity revealed by SEM analysis.

**Figure 8:**
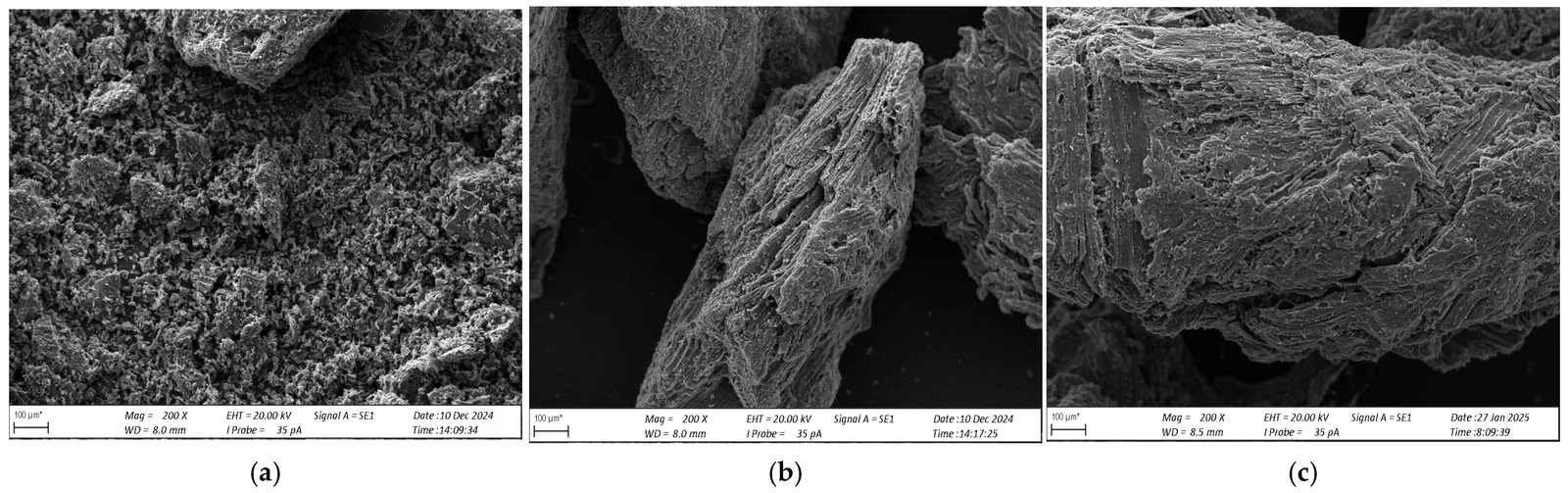
SEM images of carbonisate MDF with different particle sizes: (**a**) <500 µm, (**b**) <1000 µm, (**c**) <1500 µm.

The SEM images shown in Figure 8 were used to analyse the morphology and surface structure of the MDF carbonisate material for individual particle size fractions. The observations were carried out using a Zeiss EVO MA 15 scanning electron microscope (Carl Zeiss Microscopy GmbH, Jena, Germany). Microscopic analysis revealed clear variations in particle shape, surface area and the degree of structural heterogeneity of the carbonisate material.

Analysis of SEM images revealed clear morphological differences between the individual carbonisate fractions. The <500 µm fraction (Figure 8a) was dominated by finer and more dispersed particles with a relatively compact structure. The <1000 µm fraction (Figure 8b) was characterised by larger particles with a more developed surface area and visible layered structures. In the case of the <1500 µm fraction (Figure 8c), the largest particles were observed, with distinctly irregular morphology, a developed surface area and increased structural heterogeneity. Larger carbonisate particles may promote the formation of local inhomogeneities in the composite structure, which could contribute to the observed changes in dielectric properties.

The microstructural observations are in qualitative agreement with the trends observed in the resistivity analysis and the dielectric destabilisation index (D).

## 4. Discussion

The obtained results indicate that the granulation of MDF carbonisate was one of the main factors affecting the dielectric stability of the investigated epoxy–glass composites. An increase in carbonisate particle size resulted in a gradual decrease in surface and volume resistance, as well as an increase in the dielectric destabilization index D.

The logarithmic analysis demonstrated that the strongest reduction in dielectric properties occurred for composites containing carbonisate with a particle size of <1500 µm, particularly for the 70/30 composite system. In these materials, the highest values of ΔlogR and D were observed, indicating the greatest destabilization of insulation properties.

Optical microscopy observations confirmed that increasing the particle size of carbonisate led to higher structural heterogeneity of the composites. In the case of the <500 µm fraction, the filler particles were distributed relatively uniformly within the epoxy matrix. In contrast, composites containing <1000 µm and <1500 µm carbonisate exhibited visible agglomerates, irregular filler clusters, and local structural discontinuities. These regions may facilitate the formation of local conductive pathways and promote dielectric destabilization.

The side surface observations additionally revealed the presence of micropores and local voids between the glass reinforcement layers and the epoxy matrix. Such structural imperfections were more pronounced for larger carbonisate fractions and may contribute to local electric field concentration and partial conductivity effects.

SEM analysis of the carbonisate morphology confirmed substantial differences between the investigated granulometric fractions. The <1500 µm fraction was characterized by the most irregular morphology, highly developed surface structure, and increased heterogeneity. The presence of large irregular particles may hinder uniform dispersion in the epoxy matrix and intensify local non-uniformity within the composite structure.

The obtained results also demonstrated that surface resistance was more sensitive to the presence of carbonisate than volume resistance. This may indicate that dielectric destabilization occurred primarily within near-surface regions of the composites, where filler agglomeration and structural defects were more pronounced.

The combined electrical and microstructural analyses indicated a relationship between dielectric destabilization, filler granulation, and structural heterogeneity.

The proposed dielectric destabilization index D proved to be a useful comparative tool for assessing relative changes in the insulating properties of the investigated series of epoxy composites modified with carbonisate obtained from MDF waste.

The optical microscopy and SEM observations revealed increasing structural heterogeneity, agglomeration, and the presence of local defects with increasing carbonisate particle size. Although these observations are qualitative in nature, they remain consistent with the trends identified in the resistance analysis and the dielectric destabilization index D. Therefore, the observed microstructural features should be interpreted as factors potentially associated with the reduction in dielectric stability rather than as a quantitatively verified causal mechanism.

Compared with conventional carbon fillers such as carbon black, graphite or biochar, MDF-derived carbonisate offers the additional advantage of valorising a difficult-to-recycle waste stream. Although its morphology is more heterogeneous than that of engineered carbon fillers, the material demonstrated the ability to modify the dielectric behaviour of epoxy composites while supporting circular economy principles.

## 5. Conclusions

The particle size of MDF-derived carbonisate significantly affected the dielectric behaviour of epoxy–glass composites. An increase in carbonisate granulation resulted in a progressive reduction in the insulating properties and dielectric stability of the investigated materials.

Composites modified with carbonisate of <500 µm particle size maintained dielectric properties comparable to those of the reference materials, indicating that fine carbonisate particles can be incorporated into the polymer matrix without substantial deterioration of electrical insulation performance.

The highest dielectric destabilization was observed for the 70/30 composite containing <1500 µm carbonisate, for which the dielectric destabilization index reached D = 0.403, confirming the detrimental effect of coarse filler particles on electrical stability.

Optical microscopy and SEM observations indicated increasing structural heterogeneity and agglomeration with increasing carbonisate particle size, which may be associated with the observed reduction in dielectric stability. Larger carbonisate particles exhibited a more irregular morphology and a greater tendency toward the formation of local structural inhomogeneities, potentially affecting filler dispersion within the polymer matrix.

The results suggest that particle size had a stronger influence on dielectric stability than filler content within the investigated experimental range.

The proposed dielectric destabilization index D proved to be a useful tool for the quantitative assessment of insulation property degradation in carbonisate-modified epoxy composites.

The obtained results confirm that MDF-derived carbonisate can be used as a functional filler for epoxy composites. Appropriate control of filler particle size enables tailoring of the dielectric properties of the material while maintaining its insulating function. The investigated composites may find potential applications in structural and insulating components requiring a combination of satisfactory mechanical performance and electrical insulation, such as protective covers, housings, insulating panels, and components operating in environments where limited electrical conductivity is required. Furthermore, the utilization of MDF-derived carbonisate supports the principles of the circular economy by converting a difficult-to-recycle waste stream into a value-added raw material for sustainable polymer composite production.

This work is a step in broader research on epoxy composites modified with carbonisate obtained from waste MDF boards. Further research will focus on comprehensive characterization of the materials and assessment of their potential applications as functional composite materials in electrical engineering, electronics, and technical structures, including components of vessel equipment requiring appropriate electrical insulating and mechanical properties. In subsequent stages, it is planned to extend the characterization with studies of impedance spectroscopy, dielectric permittivity, dielectric losses and frequency-dependent properties, which will enable a more comprehensive assessment of the potential of the developed material.

## 6. Patents

The research presented in this work is associated with Polish Patent No. 248421 entitled “Sposób wytwarzania materiału kompozytowego zawierającego karbonizat, materiał kompozytowy zawierający karbonizat, zastosowanie materiału kompozytowego zawierającego karbonizat”. The patent concerns the manufacture and application of carbonisate-containing composite materials and is directly related to the materials and concepts investigated in the present study.

## Figures and Tables

**Table 1:** Composition and sample designations of composite materials prepared using the hand lay-up method.

No.	Glass Mat Layers	Resin [%]	Glass Mats Content [%]	Carbonisate [%]	Particle Size [μm]	Sample ID
1	10	65	35	0	-	X
2	10	65	30	5	<500	A
3	10	65	27.5	7.5	<500	B
4	10	65	30	5	<1000	C
5	10	65	27.5	7.5	<1000	D
6	10	65	30	5	<1500	E
7	10	65	27.5	7.5	<1500	F
8	10	60	40	0	-	Y
9	10	60	35	5	<500	G
10	10	60	32.5	7.5	<500	H
11	10	60	35	5	<1000	I
12	10	60	32.5	7.5	<1000	J
13	10	60	35	5	<1500	K
14	10	60	32.5	7.5	<1500	L
15	10	70	30	0	-	Z
16	10	70	25	5	<500	M
17	10	70	22.5	7.5	<500	N
18	10	70	25	5	<1000	O
19	10	70	22.5	7.5	<1000	P
20	10	70	25	5	<1500	R
21	10	70	22.5	7.5	<1500	S

**Table 2:** Results of measurements of surface resistance ($R_{s}$) and volume resistance ($R_{v}$) for the epoxy composites under investigation.

Sample	Designation	Carbonisate [%]	Particle Size [µm]	($R_{s}$) Surface Resistance [Ω]	($R_{v}$) Volume Resistance [Ω]
65/35	X	0	-	1 × $10^{12}$	1 × $10^{12}$
65/35	A	5	<500	1 × $10^{12}$	1 × $10^{12}$
65/35	B	7.5	<500	1 × $10^{12}$	1 × $10^{12}$
65/35	C	5	<1000	1 × $10^{12}$	(685, 852, 317) × $10^{9}$
65/35	D	7.5	<1000	1 × $10^{12}$	(420, 672, 832) × $10^{9}$
65/35	E	5	<1500	(512, 331, 357) × $10^{9}$	(689, 725, 727) × $10^{9}$
65/35	F	7.5	<1500	(427, 416, 427) × $10^{9}$	(749, 769, 750) × $10^{9}$
60/40	Y	0	-	1 × $10^{12}$	1 × $10^{12}$
60/40	G	5	<500	1 × $10^{12}$	1 × $10^{12}$
60/40	H	7.5	<500	1 × $10^{12}$	1 × $10^{12}$
60/40	I	5	<1000	1 × $10^{12}$	(721, 725, 724) × $10^{9}$
60/40	J	7.5	<1000	1 × $10^{12}$	(627, 716, 732) × $10^{9}$
60/40	K	5	<1500	(327, 456, 472) × $10^{9}$	(827, 796, 777) × $10^{9}$
60/40	L	7.5	<1500	(476, 479, 480) × $10^{9}$	(789, 787, 792) × $10^{9}$
70/30	Z	0	-	1 × $10^{12}$	1 × $10^{12}$
70/30	M	5	<500	1 × $10^{12}$	1 × $10^{12}$
70/30	N	7.5	<500	1 × $10^{12}$	1 × $10^{12}$
70/30	O	5	<1000	(456, 460, 453) × $10^{9}$	(799, 805, 802) × $10^{9}$
70/30	P	7.5	<1000	(433, 459, 449) × $10^{9}$	(801, 822, 827) × $10^{9}$
70/30	R	5	<1500	(447, 416, 467) × $10^{9}$	(747, 876, 844) × $10^{9}$
70/30	S	7.5	<1500	(435, 303, 449) × $10^{9}$	(784, 791, 843) × $10^{9}$

**Table 3:** Mean values and standard deviation (SD) surface resistance ($R_{s}$) and volume resistance ($R_{v}$) the composites under investigation.

Sample	Carbonisate [%]	Particle Size [µm]	Surface Resistance $R_{s}$ [Ω]	SD $R_{s}$ [Ω]	Volume Resistance $R_{v}$ [Ω]	SD $R_{v}$ [Ω]
65/35 X	0	–	>1 × 10^12^	–	>1 × 10^12^	–
65/35 A	5	<500	>1 × 10^12^	–	>1 × 10^12^	–
65/35 B	7.5	<500	>1 × 10^12^	–	>1 × 10^12^	–
65/35 C	5	<1000	>1 × 10^12^	–	618 × 10^9^	272 × 10^9^
65/35 D	7.5	<1000	>1 × $10^{12}$	–	641 × 10^9^	208 × 10^9^
65/35 E	5	<1500	400 × 10^9^	97.9 × 10^9^	714 × 10^9^	21.4 × 10^9^
65/35 F	7.5	<1500	423 × 10^9^	6.35 × 10^9^	756 × 10^9^	11.3 × 10^9^
60/40 Y	0	–	>1 × 10^12^	–	>1 × 10^12^	–
60/40 G	5	<500	>1 × 10^12^	–	>1 × 10^12^	–
60/40 H	7.5	<500	>1 × 10^12^	–	>1 × 10^12^	–
60/40 I	5	<1000	>1 × 10^12^	–	723 × 10^9^	2.08 × 10^9^
60/40 J	7.5	<1000	>1 × 10^12^	–	692 × 10^9^	56.6 × 10^9^
60/40 K	5	<1500	418 × 10^9^	79.5 × 10^9^	800 × 10^9^	25.2 × 10^9^
60/40 L	7.5	<1500	478 × 10^9^	2.08 × 10^9^	789 × 10^9^	2.52 × 10^9^
70/30 Z	0	–	>1 × 10^12^	–	>1 × 10^12^	–
70/30 M	5	<500	>1 × 10^12^	–	>1 × 10^12^	–
70/30 N	7.5	<500	>1 × 10^12^	–	>1 × 10^12^	–
70/30 O	5	<1000	456 × 10^9^	3.51 × 10^9^	802 × 10^9^	3.00 × 10^9^
70/30 P	7.5	<1000	447 × 10^9^	13.1 × 10^9^	817 × 10^9^	13.8 × 10^9^
70/30 R	5	<1500	443 × 10^9^	25.7 × 10^9^	822 × 10^9^	67.2 × 10^9^
70/30 S	7.5	<1500	396 × 10^9^	80.6 × 10^9^	806 × 10^9^	32.2 × 10^9^

**Table 4:** Dielectric destabilization index and classification.

Class	D Range	Interpretation
A	D = 0	no destabilization—stable insulator
B	0 < D ≤ 0.25	slight destabilization
C	0.25 < D ≤ 0.50	moderate destabilization
D	D > 0.50	strong destabilization/possible local percolation

**Table 5:** Values of logarithmic resistance parameters and dielectric destabilization indices of the analysed epoxy composites.

Sample	Sample ID	log10(*R_s_*)	log10(*R_v_*)	Δlog*R_s_*	Δlog*R_v_*	SI *R_s_*	SI *R_v_*	D	Class
65/35	X	12	12	0	0	1	1	0	A
65/35	A	12	12	0	0	1	1	0	A
65/35	B	12	12	0	0	1	1	0	A
65/35	C	12	11.791	0	0.209	1	0.618	0.209	B
65/35	D	12	11.807	0	0.193	1	0.641	0.193	B
65/35	E	11.602	11.853	0.398	0.147	0.400	0.714	0.398	C
65/35	F	11.627	11.879	0.373	0.121	0.423	0.756	0.373	C
60/40	Y	12	12	0	0	1	1	0	A
60/40	G	12	12	0	0	1	1	0	A
60/40	H	12	12	0	0	1	1	0	A
60/40	I	12	11.859	0	0.141	1	0.723	0.141	B
60/40	J	12	11.840	0	0.160	1	0.692	0.160	B
60/40	K	11.622	11.903	0.378	0.097	0.418	0.800	0.378	C
60/40	L	11.68	11.897	0.32	0.103	0.478	0.789	0.32	C
70/30	Z	12	12	0	0	1	1	0	A
70/30	M	12	12	0	0	1	1	0	A
70/30	N	12	12	0	0	1	1	0	A
70/30	O	11.659	11.904	0.341	0.096	0.456	0.802	0.341	C
70/30	P	11.650	11.912	0.350	0.088	0.447	0.817	0.350	C
70/30	R	11.647	11.915	0.353	0.085	0.443	0.822	0.353	C
70/30	S	11.597	11.906	0.403	0.094	0.396	0.806	0.403	C

**Table 6:** Mean values of logarithmic resistance parameters and the dielectric destabilization index D depending on carbonisate particle size.

Particle Size [µm]	Mean log10(*R_s_*)	Mean log10(*R_v_*)	Mean D
<500	12	12	0
<1000	11.885	11.852	0.232
<1500	11.629	11.892	0.371

**Table 7:** Mean values of logarithmic resistance parameters and the dielectric destabilization index D depending on carbonisate content.

Caronisate [%]	Mean log10(*R_s_*)	Mean log10(*R_v_*)	Mean D
5	11.837	11.914	0.202
7.5	11.839	11.916	0.200

**Table 8:** Mean values of logarithmic resistance parameters and the dielectric destabilization index D for individual composite compositions.

Compositions	Mean log10(*R_s_*)	Mean log10(*R_v_*)	Mean D
65/35	11.872	11.888	0.196
60/40	11.884	11.916	0.166
70/30	11.759	11.940	0.241

## Data Availability

The original contributions presented in this study are included in the article. Further inquiries can be directed to the corresponding author.
